# pyBadlands: A framework to simulate sediment transport, landscape dynamics and basin stratigraphic evolution through space and time

**DOI:** 10.1371/journal.pone.0195557

**Published:** 2018-04-12

**Authors:** Tristan Salles, Xuesong Ding, Gilles Brocard

**Affiliations:** School of Geosciences, University of Sydney, Sydney, NSW, 2006, Australia; Texas A&M University System, UNITED STATES

## Abstract

Understanding Earth surface responses in terms of sediment dynamics to climatic variability and tectonics forcing is hindered by limited ability of current models to simulate long-term evolution of sediment transfer and associated morphological changes. This paper presents pyBadlands, an open-source python-based framework which computes over geological time (1) sediment transport from landmasses to coasts, (2) reworking of marine sediments by longshore currents and (3) development of coral reef systems. pyBadlands is cross-platform, distributed under the GPLv3 license and available on GitHub (http://github.com/badlands-model). Here, we describe the underlying physical assumptions behind the simulated processes and the main options already available in the numerical framework. Along with the source code, a list of hands-on examples is provided that illustrates the model capabilities. In addition, pre and post-processing classes have been built and are accessible as a companion toolbox which comprises a series of workflows to efficiently build, quantify and explore simulation input and output files. While the framework has been primarily designed for research, its simplicity of use and portability makes it a great tool for teaching purposes.

## Introduction

Over the last decades, many numerical models have been proposed to simulate how the Earth surface has evolved over geological time scales in response to different driving forces such as tectonics or climatic variability [1–5]. These models combine empirical data and conceptual methods into a set of mathematical equations that can be used to reconstruct landscape evolution and associated sediment fluxes [6, 7]. They are currently used in many research fields such as hydrology, soil erosion, hillslope stability and general landscape studies.

Numerous models have focused on stream bed dynamics and erosion [7–11]. Much less work has been devoted to simulate regional to continental sediment deposition and associated sedimentary basin architecture [6, 12]. With a few exceptions [13–15], most of these models have either focused on one part of the sediment routing system (e.g., fluvial geomorphology, coastal erosion, carbonate platform development), or captured the entire routing within a few simple laws commonly derived from diffusion-based equations, well suited to low, pluri-kilometric spatial resolution [16]. These limitations often restrict our understanding of sediment fate from source to sink. It also makes it difficult to link site-specific observations to numerical model outputs.

**Fig 1:**
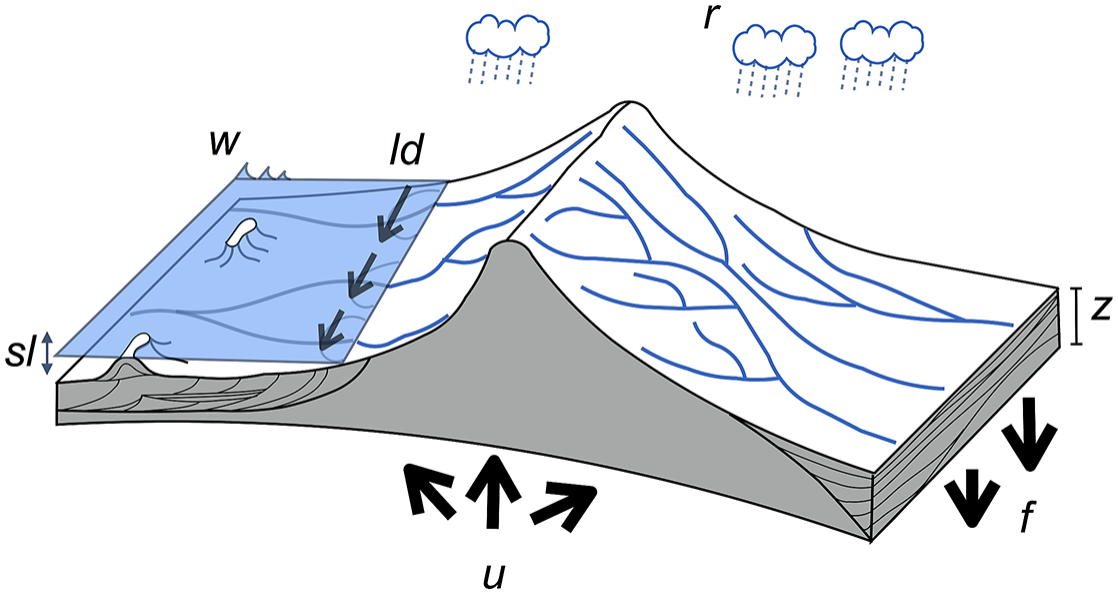
A schematic of our model illustrating the main variables and forcing conditions. **w** represents the wave boundary conditions, **ld** the longshore drift, **sl** the sea-level, **u** the tectonic, **f** the flexural isostasy and **r** the rainfall patterns. The stratigraphic evolution and morphology are computed through time.

The framework and the development efforts presented in this paper are intended to address these shortcomings. It provides a more direct and flexible way to analyse inter-connectivities between land, marine and reef environments, in that it explicitly links these systems together (Fig 1). pyBadlands is a python-friendly version of Badlands [12, 17] which provides a programmable and flexible front end to all the previous functionality of this code and adds new surface process simulation capability, portability and usability.

Landscape dynamics in our model are driven by a series of stream power incision laws that are in some sense an over-simplification of natural phenomenon [18]. This becomes apparent at fine scales on which hillslope diffusion becomes important (*e.g.* total basin area under 10 km^2^) [19]. Therefore pyBadlands is mainly designed to study problems over regional to continental scale and over geological time (10^3^-10^7^ years). Due to the spatial resolution, our model is not intended to look at the evolution of individual fluvial channels but rather to the large scale, long term evolution of an overall landscape. Following a similar approach, the simulation of waves and carbonate platform rely on some simple mathematical formulations (linear wave theory and parametric laws based on fuzzy logic for the carbonates) that do not capture the complex physical and biological interactions known to take place in the natural systems. As such our model is not intended for engineering applications but falls into the so-called reduced-complexity family of geological models in that it relies on simple, though not simplistic physics to drive fluid motion and associated sediment transport by rivers and waves [16, 20]. Engineering models are expected to work at relatively high accuracy and are often used to predict short-term evolution of a particular physical process in which initial and boundary conditions are well known because they can be measured today. On the other hand, geological models, such as pyBadlands, operate over periods of thousands to millions of years [12] where initial conditions are not known in detail, and for which first-order evaluation of landscape erosion, sediment accumulation or current velocities are tolerable. Therefore the model described in this paper is meant for geological applications and primarily focused on the description of Earth surface evolution and sedimentary basins over large spatial and temporal scales. Even though the primary goal of the proposed framework is intended to look at these long-term and regional scale processes, the open-source nature of the code make it possible to implement more accurate approaches to describe smaller temporal and spatial scale processes.

We illustrate in an example applied to a mixed siliciclastic-carbonate system how pyBadlands can be used to study: sediment transport from landmasses to coasts, reworking of marine sediments by longshore currents, and development of coral reef systems.

## Simulated processes

### Fluvial system

**Fig 2:**
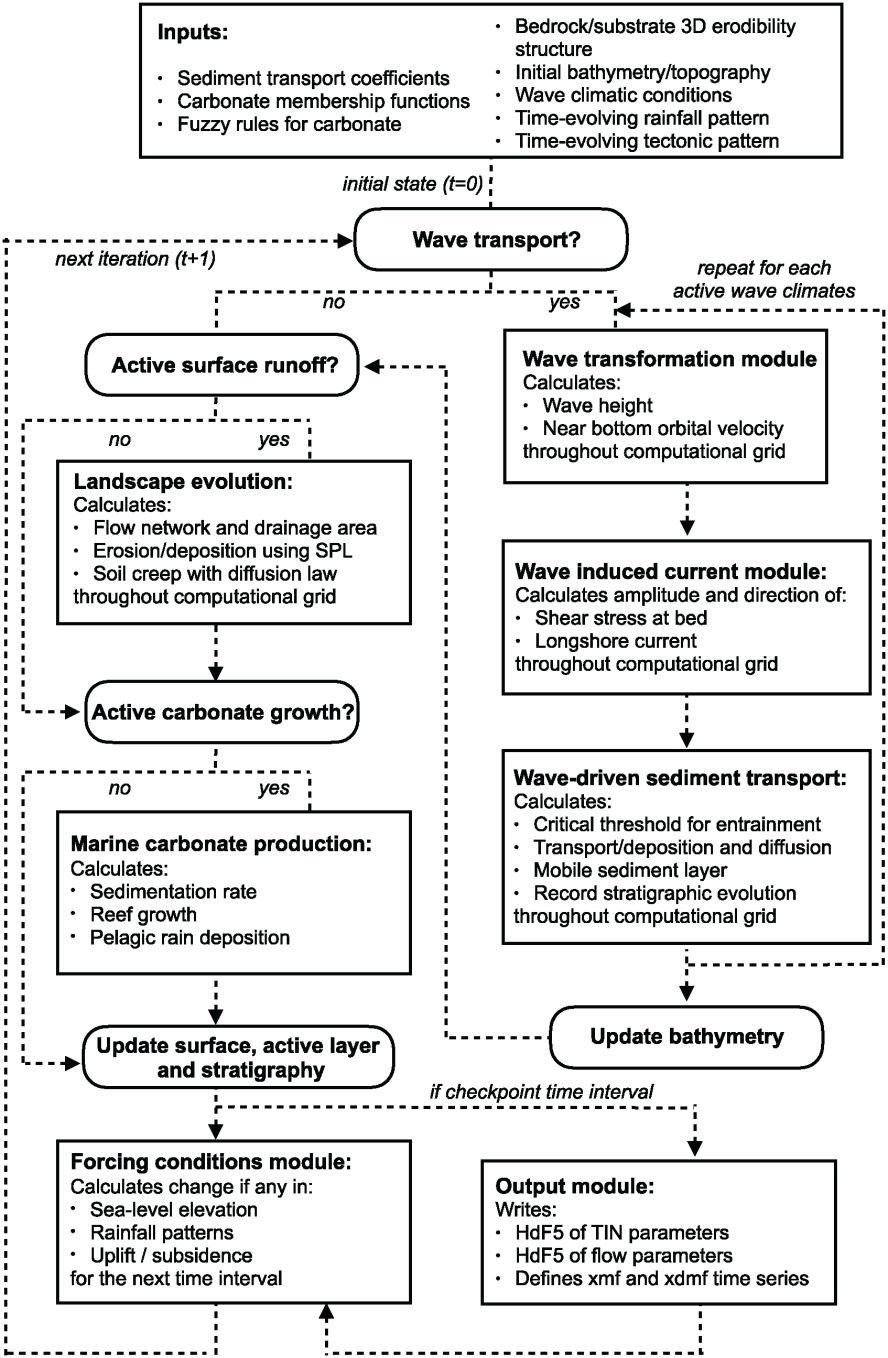
Main components of pyBadlands workflow.

pyBadlands uses a triangular irregular network (TIN) to solve the geomorphic equations presented below [21]. The continuity equation is defined using a finite volume approach and relies on the method described in Tucker et al. [22]. Fig 2 illustrates the relationships between the different components of the framework.

To solve channel incision and landscape evolution, the algorithm follows the O(n)-efficient ordering method from Braun and Willett [23] and is based on a single-flow-direction (SFD) approximation assuming that water goes down the path of the steepest slope [24].

**Fig 3:**
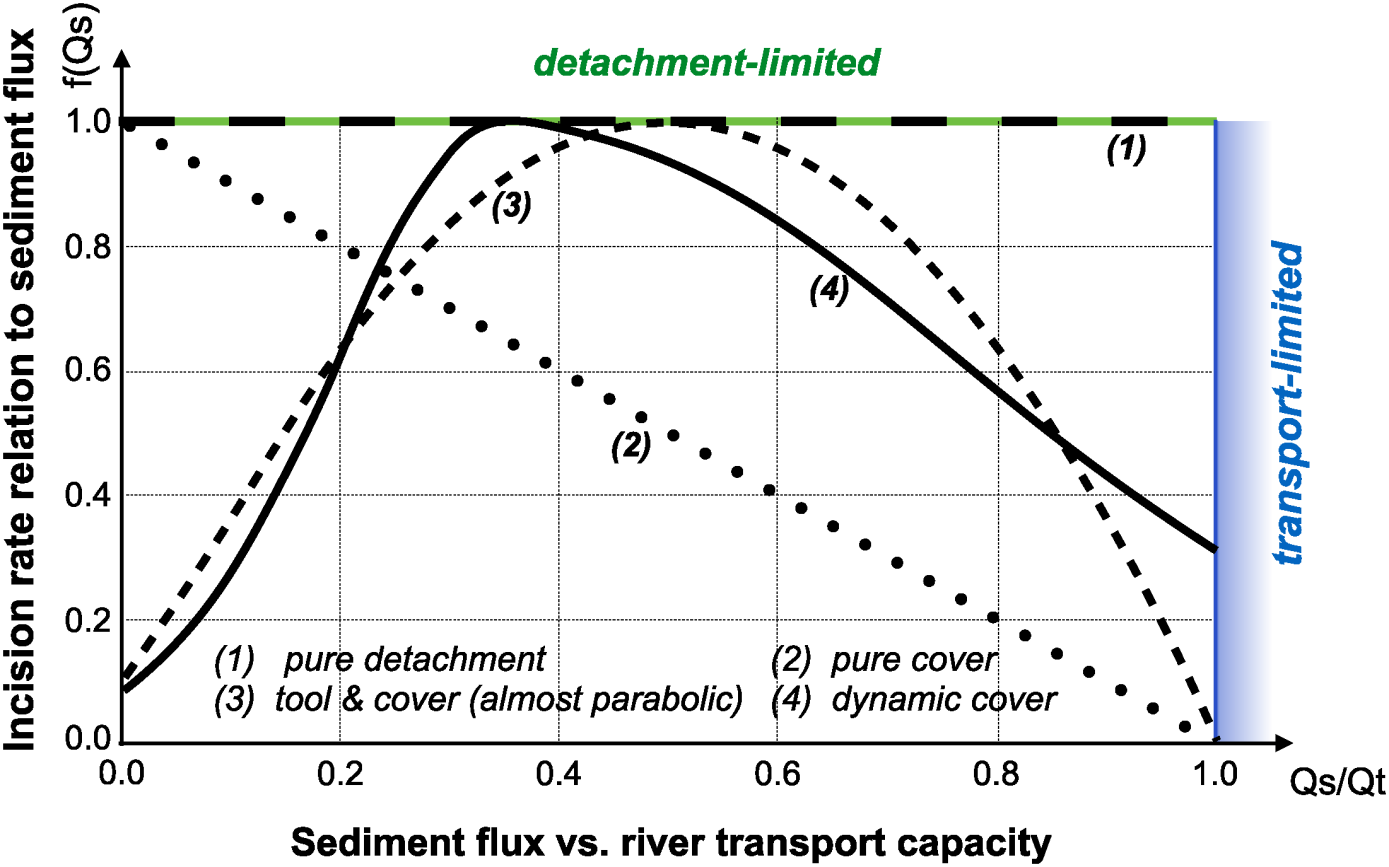
Model space for implemented stream power-based incision laws. It shows the dependence of river incision rate on sediment flux (adapted from Hobley et al. [7]).

Several formulations of river incision have been proposed to account for long term evolution of fluvial system [2, 25]. These formulations describe different erosional behaviours ranging from detachment-limited incision, governed by bed resistance to erosion, to transport-limited incision, governed by flow capacity to transport sediment available on the bed. Mathematical representation of erosion processes in these formulations is often assumed to follow a stream power law [15]. These relatively simple approaches have two main advantages. First, they have been shown to approximate the first order kinematics of landscape evolution across geologically relevant timescales (>10^4^ years). Second, neither the details of long term catchment hydrology nor the complexity of sediment mobilisation dynamics are required. However, other formulations are sometimes necessary when addressing specific aspects of landscape evolution. Below, we present the six incision laws currently available in pyBadlands (Fig 3).

#### Detachment-limited model

**Fig 4:**
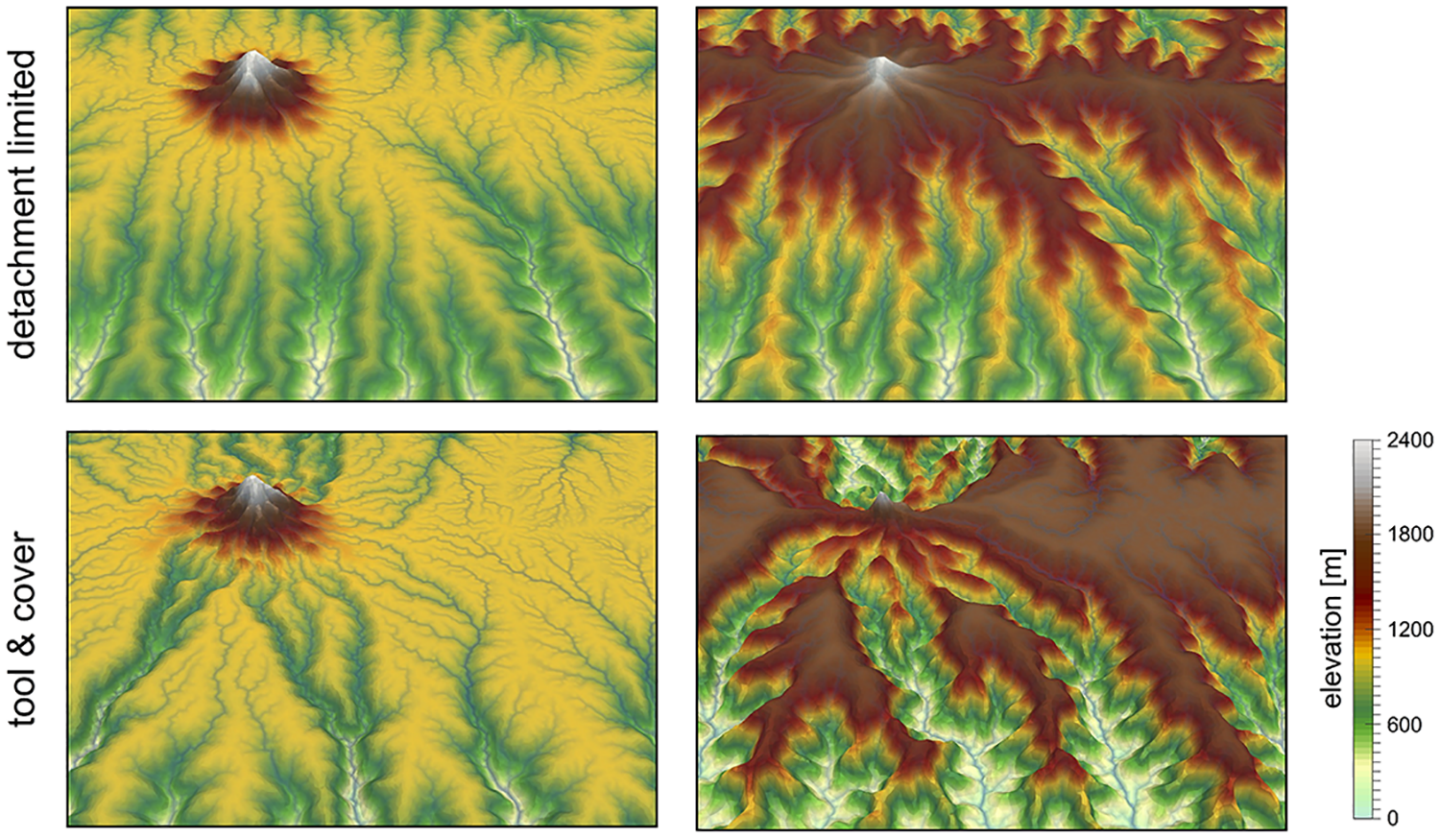
Illustration of the impact of detachment versus transport limited (tool & cover option 3) formulations on landscape dynamics. Evolution of dissection of an uplifting landscape composed of a flat surfaces dotted with an isolated peak, after 5 and 9 Ma of dissection. The modeling shows how the abundant bedload shed by the isolated peak boosts incision along the receiving streams (tool effect).

The default pyBadlands fluvial incision is based on the detachment-limited stream power law (Fig 3—option 1, Fig 4), in which erosion rate $\dot{\epsilon }$ depends on drainage area *A*, net precipitation *P* and local slope *S* and takes the form:

$$\begin{array}{c} \dot{\epsilon }=\kappa_{d}P^{l}{\left(PA\right)}^{m}S^{n} \end{array}$$ (1)

*κ*_*d*_ is a dimensional coefficient describing the erodibility of the channel bed as a function of rock strength, bed roughness and climate, *l*, *m* and *n* are dimensionless positive constants. Default formulation assumes *l* = 0, *m* = 0.5 and *n* = 1. The precipitation exponent *l* allows for representation of climate-dependent chemical weathering of river bed across non-uniform rainfall [26]. In this model sediment deposition occurs solely in topographically closed depression and offshore.

#### Transport-limited models

Here, volumetric sediment transport capacity (*Q*_*t*_) is defined using a power law function of unit stream power:

$$\begin{array}{c} Q_{t}=\kappa_{t}{\left(PA\right)}^{m_{t}}S^{n_{t}} \end{array}$$ (2)

where *κ*_*t*_ is a dimensional coefficient describing the transportability of channel sediment and *m*_*t*_ and *n*_*t*_ are dimensionless positive constants. In Eq (2), the threshold of motion (the critical shear stress) is assumed to be negligible [27].

An additional term is now introduced in the stream power model:

$$\begin{array}{c} \dot{\epsilon }=\kappa f\left(Q_{s}\right){\left(PA\right)}^{m}S^{n} \end{array}$$ (3)

with *f*(*Q*_*s*_) representing a variety of plausible models for the dependence of river incision rate on sediment flux *Q*_*s*_ (Fig 3). In the standard detachment-limited, *f*(*Q*_*s*_) is equal to unity, which corresponds to cases where *Q*_*s*_ ≪ *Q*_*t*_. All sediment is dispersed downstream and the incision limiting factor is bedrock erodibility. Addition of the transport-limited function results in the fact that, where sediment flux equals or exceeds transport capacity (*Q*_*s*_/*Q*_*t*_ ≥ 1) the system becomes transport-limited and depositional if *Q*_*s*_/*Q*_*t*_ > 1. In this model the time-evolving distribution of erosion and sedimentation, is affected by the distribution of detachment-limited and transport-limited reaches, which is controlled by the respective values of *κ*_*d*_ and *κ*_*t*_.

The transition from one behavior to the other can be treated either abruptly, progressively, through the use of one of the following formulations:

**Linear decline** This model belongs to the undercapacity family of models: it assumes that stream incision represents the expenditure of the energy in excess of that needed to transport the bypassing sediment load. Stream incision potential decreases linearly from a maximum where sediment flux is negligible, to zero where sediment flux equals transport capacity (Fig 3—option 2). Conceptually, this law mimics the transfer of stream energy from erosion to transport processes [1]:

$$\begin{array}{c} f\left(Q_{s}\right)=1-\frac{Q_{s}}{Q_{t}} \end{array}$$ (4)

**Almost parabolic** Both qualitative and experimental observations have shown that sediment flux has a dual role in the river bed incision. First, when sediment flux is low relative to carrying capacity, erosion potential increases with sediment flux (tool effect: bedrock abrasion and plucking). Then, with increased sediment flux, erosion is inhibited (cover effect: sediments protect the bed from impacts by saltating particles). Following Gasparini et al. [28], we adopt a parabolic form that reaches a maximum at *Q*_*s*_/*Q*_*t*_ = 1/2 [29, 30] (Fig 3—option 3, Fig 4):

$$\begin{array}{c} f\left(Q_{s}\right)=\left\{ \begin{array}{cc} 1-4{\left(\frac{Q_{s}}{Q_{t}}-0.5\right)}^{2} & \text{if}\;Q_{s}/Q_{t}>0.1\\ 2.6\frac{Q_{s}}{Q_{t}}+0.1 & \text{if}\;Q_{s}/Q_{t}<0.1 \end{array}\right. \end{array}$$ (5)

**Dynamic cover** Typically gravel-river beds have an armoured layer of coarse grains on the surface, which acts to protect finer particles underneath from erosion. To account for sediment and spatial heterogeneity in the armouring of the river bed, Turowski et al. [11] proposed a modified form of the ‘almost parabolic’ model that better estimates the original Sklar and Dietrich [10] experiments (Fig 3—option 4). It takes the form of two combined Gaussian functions:

$$\begin{array}{c} f\left(Q_{s}\right)=\exp\left[-{\left(\frac{Q_{s}/Q_{t}-0.35}{C_{h}}\right)}^{2}\right] \end{array}$$ (6)

where *C*_*h*_ is set to 0.22 for *Q*_*s*_/*Q*_*t*_ ≤ 0.35 and 0.6 when *Q*_*s*_/*Q*_*t*_ > 0.35.

**Saltation abrasion** Sklar & Dietrich [29, 31] were the first who proposed also a formulation for tool and cover mechanisms which relates bedrock incision to abrasion of saltating bed load. The expression shares the same form as the sediment flux-dependent incision rule presented by Whipple & Tucker [1] (Eq 3) with significantly different exponent values:

$$\begin{array}{c} \dot{\epsilon }=\kappa_{sa}f\left(Q_{s}\right)A^{-0.25}S^{-0.5} \end{array}$$ (7)

in which the dependence of incision rate to sediment flux is defined as:

$$\begin{array}{c} f\left(Q_{s}\right)=\frac{Q_{s}}{W}\left(1-\frac{Q_{s}}{Q_{t}}\right) \end{array}$$ (8)

where *W* represents the channel width and is expressed as a power law relation between width and discharge *W* = *κ*_*w*_ *A*^*b*^.

**Abrasion incision** Parker [32] presented an incision model in which two processes dominate erosion of the channel bed: plucking of bedrock blocks and abrasion by saltating bed load. The approach takes the following form:

$$\begin{array}{c} \dot{\epsilon }=\kappa_{ai}\frac{Q_{s}}{W}\left(1-\frac{Q_{s}}{Q_{t}}\right) \end{array}$$ (9)

where the only difference with the saltation-abrasion model is in the exponents on both the discharge and slope which are set to zero.

### Hillslope processes

**Fig 5:**
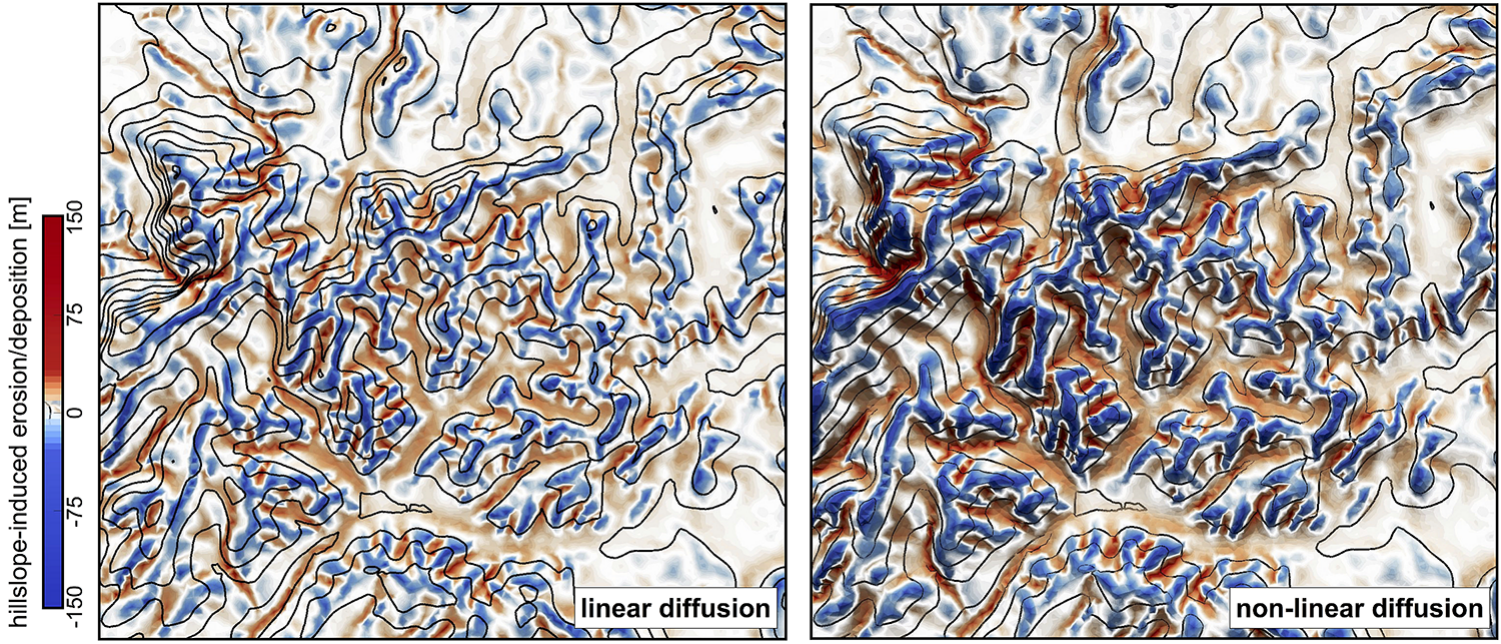
Erosion/deposition induced after 130 ka of hillslope diffusion using the linear and non-linear formulations. Left: Linear diffusion produces convex upward slopes (*κ*_*hl*_ = *κ*_*hn*_ = 0.05). Right: non-linear approach tends to have convex to planar profiles as hillslope processes dominate when slopes approach or exceed the critical slope (*S*_*c*_ = 0.8) [33, 34].

Along hillslopes, we state that the flux of sediment is proportional to the gradient of topography (Fig 5).

One can choose between two options to simulate these processes. In the first, we use a linear diffusion law commonly referred to as soil creep [35, 36]:

$$\begin{array}{c} \frac{\partial z}{\partial t}=\kappa_{hl}\nabla^{2}z \end{array}$$ (10)

in which *κ*_*hl*_ is the diffusion coefficient and can be defined with different values for the marine and land environments. It encapsulates, in a simple formulation, a variety of processes operating over short ranges on the superficial veneer of soils and sediments. *κ*_*hl*_ varies, no exhaustively, as a function of substrate, lithology, soil depth, climate and biological activity.

Field evidence, however, suggests that the linear diffusion approximation (Eq 10) is only rarely appropriate [33, 34, 37, 38]. Instead, Andrews and Bucknam [39] and Roering et al. [40, 41] proposed a non-linear formulation of diffusive hillslope transport, assuming that flux rates increase to infinity if slope values approach a critical slope *S*_*c*_. This alternative formulation is available as a second option and takes the following form in our model:

$$\begin{array}{c} \frac{\partial z}{\partial t}=\nabla \cdot \frac{\kappa_{hn}\nabla z}{1-\left(|\nabla z\right|/S_{c}{)}^{2}} \end{array}$$ (11)

### Wave-induced longshore drift

We adopt the most basic known principles of wave motion, *i.e.*, the linear wave theory, to simulate wave-induced longshore drift [42, 43]. Wave celerity *c* is governed by:

$$\begin{array}{c} c=\sqrt{\frac{g}{\kappa }\tanh\kappa d} \end{array}$$ (12)

where *g* is the gravitational acceleration, *κ* the radian wave number (equal to 2*π*/*L*, with *L* the wave length), and *d* is the water depth. In deep water, the celerity is dependent only on wave length ($\sqrt{gL/2\pi }$); in shallow water, it depends on depth ($\sqrt{gd}$). From wave celerity and wave length, we calculate wave front propagation (including refraction) using the Huygens principle [20]. From this, we deduce the wave travel time and define wave directions from lines perpendicular to the wave front. Wave height (*H*) is then calculated along wave front propagation. The algorithm takes into account wave energy dissipation in shallow environment as well as wave-breaking conditions.

Wave-induced sediment transport is related to the maximum bottom wave-orbital velocity *u*_*w*,*b*_. Assuming the linear shallow water approximation [44], its expression is simplified as:

$$\begin{array}{c} u_{w,b}=\left(H/2\right)\sqrt{g/d} \end{array}$$ (13)

Under pure waves (*i.e.*, no superimposed current), the wave-induced bed shear stress *τ*_*w*_ is typically defined as a quadratic bottom friction [45]:

$$\begin{array}{c} \tau_{w}=\frac{1}{2}\rho f_{w}u_{w,b}^{2} \end{array}$$ (14)

with *ρ* the water density and *f*_*w*_ is the wave friction factor. Considering that the wave friction factor is only dependent of the bed roughness *k*_*b*_ relative to the wave-orbital semiexcursion at the bed *A*_*b*_ [46], we define:

$$\begin{array}{c} f_{w}=1.39{\left(A_{b}/k_{b}\right)}^{-0.52} \end{array}$$ (15)

where *A*_*b*_ = *u*_*w*,*b*_*T*/2*π* and *k*_*b*_ = 2*πd*_50_/12, with *d*_50_ median sediment grain-size at the bed and *T* the wave period.

For each wave condition, the wave transformation model computes wave characteristics and the induced bottom shear stress. These parameters are subsequently used to evaluate the long-term sediment transport active over the simulated region.

We assume that flow circulation is mainly driven by waves, while other processes such as coastal upwelling, tidal, ocean or wind-driven currents are ignored. The proposed method consists in producing *snapshots* of wave-driven circulation distribution resulting from series of deep-water wave scenarios by computing time-averaged longshore current. In nearshore environments, longshore current runs parallel to the shore and significantly contributes to sediment transport [47, 48]. The longshore current velocity ($\vec{v_{l}}$) in the middle of the breaking zone is defined by [49]:

$$\begin{array}{c} \vec{v_{l}}=\kappa_{l}u_{w,b}cos\left(\theta \right)sin\left(\theta \right)\vec{k} \end{array}$$ (16)

with *θ* the angle of incidence of the incoming waves, *κ*_*l*_ a scaling parameter and $\vec{k}$ the unit vector parallel to the breaking depth contour.

**Fig 6:**
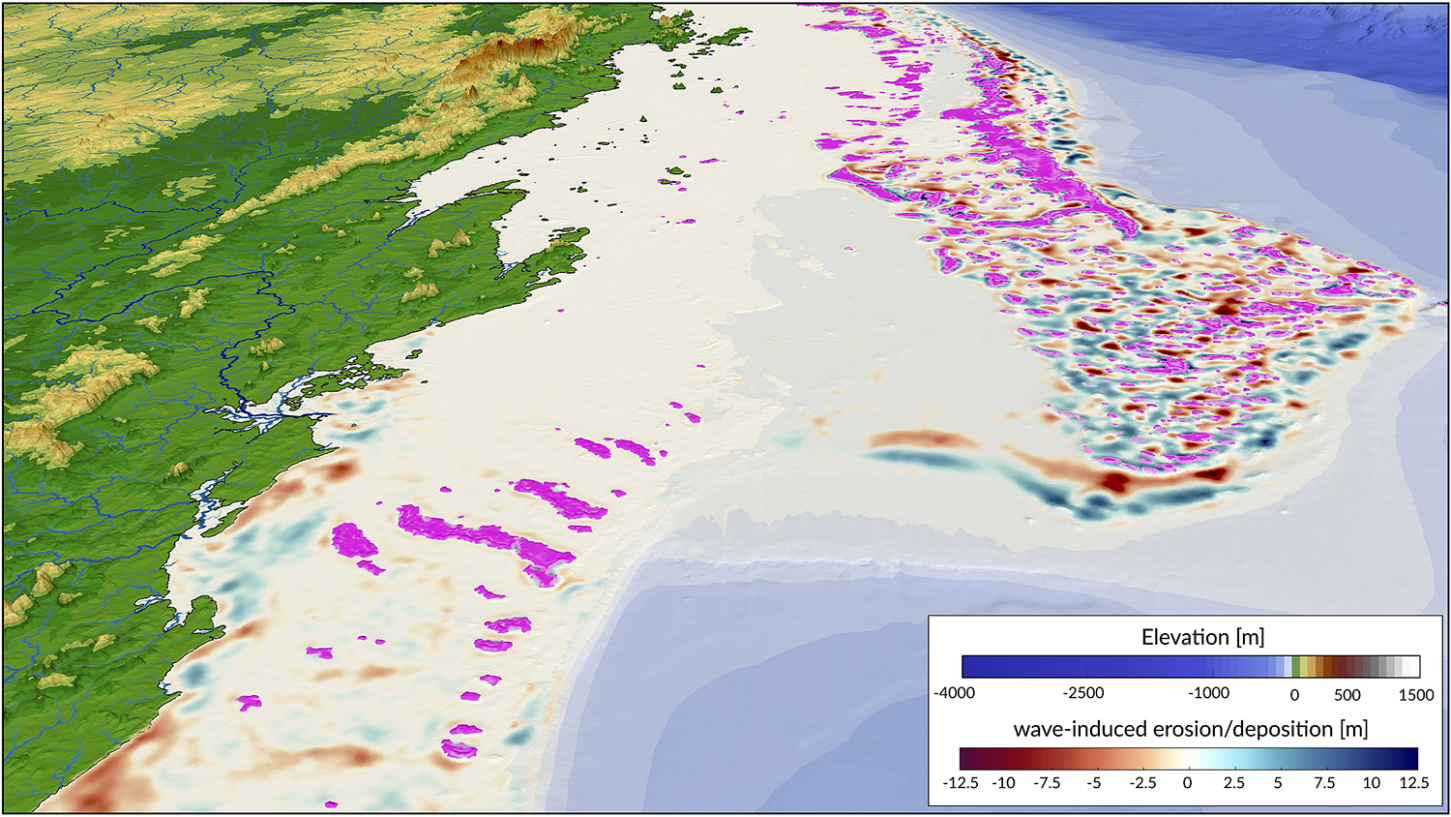
Example of cumulative wave-induced erosion/deposition during a transgression event in the southern portion of the Great Barrier Reef (simulated time: 14 ka). Wave-induced shear stress and associated longshore sediment transport are evaluated every 50 years. Pink patches show location of produced coral reefs.

In regions where wave-induced shear stress (Eq 14) is greater than the critical shear stress [50] derived from the Shields parameter (*τ*_*c*_ = *θ*_*c*_*gd*_50_(*ρ*_*s*_ − *ρ*_*w*_)), bed sediments are entrained. The erosion thickness *h*_*e*_ is limited to the top sedimentary layer and for simplicity is assumed to follow a logarithmic form [51]:

$$\begin{array}{c} h_{e}=C_{e}\ln\left(\tau_{w}/\tau_{c}\right)\;\text{where}\;\tau_{w}>\tau_{c} \end{array}$$ (17)

where *C*_*e*_ is an entrainment coefficient controlling the relationship between shear stress and erosion rate [52]. Once entrained, sediments are transported following the direction of longshore currents and are deposited in regions where *τ*_*w*_ < *τ*_*c*_ [53] (Fig 6).

### Carbonate production

The organisation of coral reef systems is known to be large and complex and we are still limited in our understanding of their temporal and spatial evolution [54]. Additionally, most datasets of carbonate systems are often descriptive, context-dependent, or based on measurements with large uncertainties. Alternative approaches, such as fuzzy logic or cellular automata algorithms, have proven to be viable options to simulate these types of system [55–57]. Fuzzy logic methods are able to create logical propositions from qualitative data by using linguistic logic rules and *fuzzy sets* [58]. These fuzzy sets are defined with continuous boundaries rather than *crisp* discontinuous ones often used in conventional methods [59].

**Fig 7:**
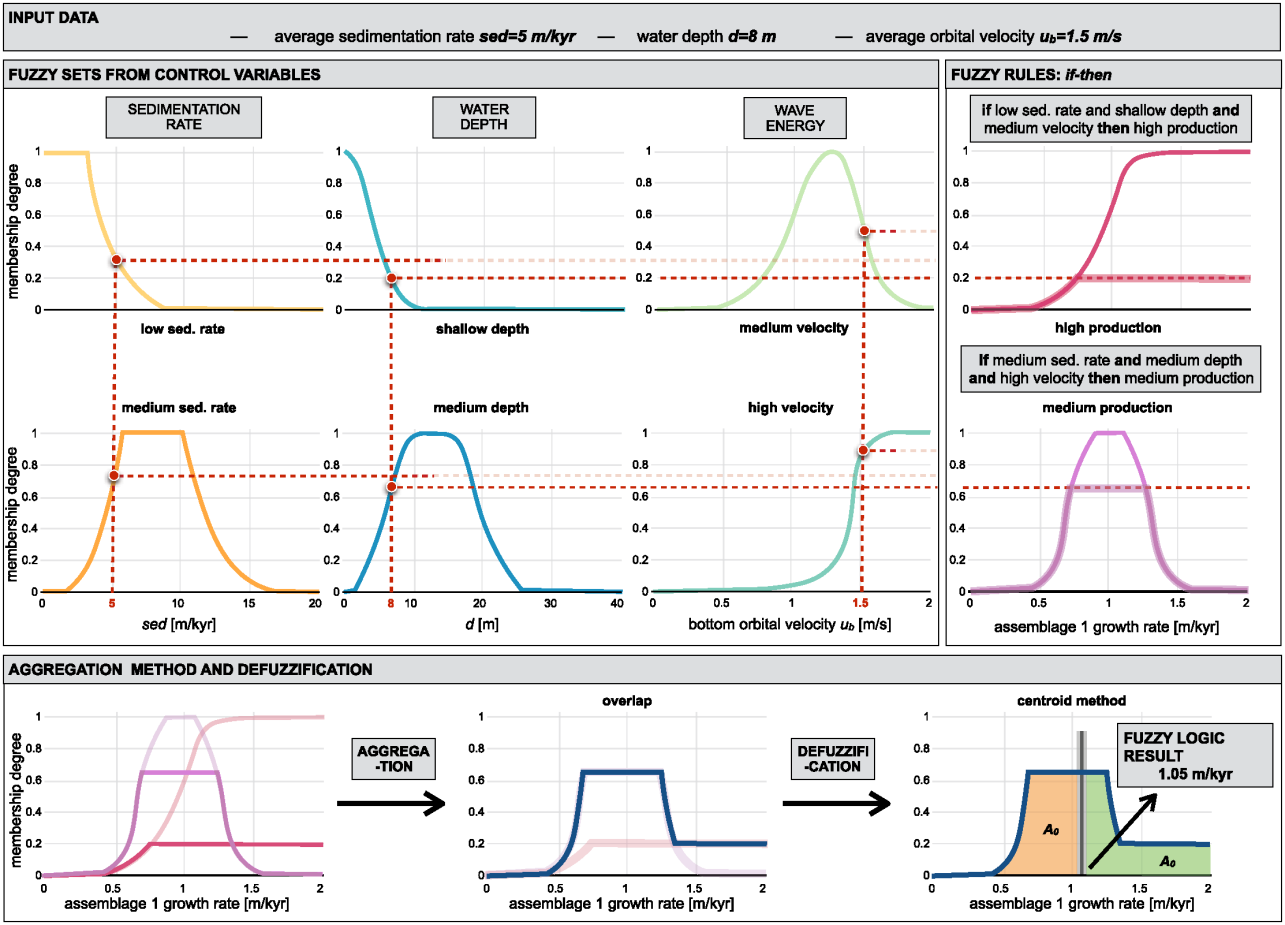
Diagram of fuzzy logic process used to evaluate a specific coral assemblage growth rate. Coral assemblage refers to a specific coral colony or community often derived from depth zonation.

Based on a fuzzy logic approach, carbonate system evolution in pyBadlands is driven entirely by a set of rules which variables are fully adjustable. The utility and effectiveness of the approach is mostly based on the user’s understanding of the modelled carbonate system [14]. The technique is specifically useful to understand how particular variables influence carbonate depositional geometries and reef adaptation (Fig 7).

Here, carbonate growth depends on three types of control variables: depth (or accommodation space), wave energy (derived from ocean bottom orbital velocity) and sedimentation rate. For each of these variables, one can define a range of fuzzy sets using membership functions [58]. A membership function is a curve showing the degree of truth (*i.e.* ranging between 0 and 1) of membership in a particular fuzzy set (Fig 7). These curves can be simple triangles, trapezoids, bell-shaped curves, or display more complicated shapes as shown in Fig 7.

Production of any specific coral assemblage is then computed from a series of fuzzy rules. A fuzzy rule is a logic *if-then* rule defined from a fuzzy sets [55]. Here, the combination of the fuzzy sets in each fuzzy rule is restricted to the *and* operator. The amalgamation of competing fuzzy rules is usually referred to as a fuzzy system. Summation of multiple rules from the fuzzy system by truncation of the membership functions produces a fuzzy answer in the form of a membership set (Fig 7). The last step consists in computing a single number for this fuzzy set through *defuzzification* [60]. In our approach, the centroid (centre of gravity) for the area below the membership set is taken as the *defuzzified* output value. The process returns a *crisp* value of coral assemblage growth on each cell of the simulated region (Fig 6).

### Extrinsic forcings

Over geological time scales, sediment transport from source to sink is primarily controlled by climate, tectonics and eustatism (Fig 1). In pyBadlands, the following set of external forcing mechanisms could be considered: (i) sea-level fluctuations, (ii) subsidence, uplift and horizontal displacements, (iii) rainfall regimes and (iv) boundary wave conditions.

Tectonic forcing is driven by a time series of tectonic deformation maps. Each map can provide its own specific spatial cumulative displacements making it possible to simulate complex 3D tectonic evolution in both vertical (uplift and subsidence) and horizontal directions. When 3D deformations are imposed, the surface is first advected by tectonic forces before being modified by surface processes. Tectonic advection affects the density of the nodes evolves over time, which leads to uneven resolution, with places showing either rarefaction or accumulation of nodes. To limit this effect, the advected surface is resampled by adding or removing nodes to ensure homogeneous nodes distribution. The model uses the node refinement technique proposed by Thieulot et al. [61] to perform this transformation.

Sea-level curves (sea-level elevation through time) can be imposed from either a published eustatic curve [62, 63] or directly defined by the user.

Temporal variations in precipitation may be applied either as a constant values (metres per year) or a set of maps representing spatially changing rainfall regimes. In addition, to account for the interactions between rainfall and topography, orographic precipitation can be modeled from the topographic surface using the linear model of Smith & Barstad [64]. Coupled evolution of precipitation patterns and topography can be use to quantify the relative importance of climate, erosion and tectonic in mountain geomorphology.

To evaluate marine sediment transport over several thousands of years, the approach taken here relies on a stationary representation of prevailing fair-weather wave conditions. The wave transformation model is generally performed for time intervals varying from 5 to 50 years. The aim is to simulate realistic wave fields by imposing a sequence of wave forcing conditions. At any given time interval, we define a percentage of activity for each deep-water wave conditions as well as a significant wave height. Then, the bathymetry is used to compute associated wave parameters.

The forcing mechanisms described above will directly control the evolution of sediment transport, associated stratal architecture as well as carbonate production.

## Usability

### Portability

pyBadlands is an open-source package distributed under GNU GPLv3 license. The source code is available on GitHub (http://github.com/badlands-model). Structurally the code is a python front end with a C and Fortran middle layer to efficiently compute some of the heaviest functions. This python-friendly version provides a programmable and flexible interface which maximises its portability across platforms.

Instructions to install the code and the associated dependencies on a local system are provided on our wiki pages (https://github.com/badlands-model/pyBadlands/wiki) along with model options and a series of hands-on examples.

The easiest way to use pyBadlands is via our Docker container (searching for pybadlands-demo-dev—https://hub.docker.com/u/badlandsmodel/, on Kitematic) which is shipped with the complete list of dependencies, the model companion and the examples. Models data and outputs ran from within the container will not persist when that container is no longer active. To provide better interfacing between the container and the host filesystem, pyBadlands image can be mounted on a local volume which allow for easy access and ability to store securely model results.

### Interactions with other packages

pyBadlands main calculations are performed on a TIN. However the code creates its own Delaunay triangulation using the Shewchuk’s Triangle library [65] from regularly defined input datasets (e.g., topography grid, rain maps, tectonic maps). In that way, users provide standard regularly spaced ASCII datasets. The only requirement is to follow a specific column-major order for the declaration of each nodes values which is consistent between imported datasets starting from the south-west and ending on the north-east corner.

Model results consist of time series of surface evolution, river and catchment dynamics grids as well as underlying stratigraphic architecture mesh. These outputs are all produced as Hdf5 binary files making it possible to interact with multiple existing visualisation and analysis software, such as Paraview or MayaVi.

Initial initial surfaces have UTM coordinates. Functions have been added to easily extract Web Map Service dataset (one example is provided to illustrate how to define an initial topography grid from ETOPO5 datasets). Hdf5 files can also be quickly converted to other conventional raster GIS file formats such as ASCII grids.

The gFlex modular python package [66] has been integrated as a component in pyBadlands to incorporate the effects of flexural isostasy on basin stratgraphic architecture. It allows to compute isostatic deflections of the Earth’s lithosphere for uniform or nonuniform flexural rigidity. Surface loads affect the erosion/deposition patterns associated to modelled surface processes.

### Hands-on examples

A series of examples have been made available with the source code. They illustrate the different capabilities of our package and are an informative starting point to learn how to use pyBadlands. Each example is packaged into a specific folder. Each folder includes (1) an input XML file where the different options and default values for the considered experiment are set, (2) a *data* folder containing the initial surface and potentially some forcing files (e.g. sea-level, rainfall or tectonic grids) and (3) a series of IPython notebooks used to run the experiment and perform some pre or post-processing tasks.

These examples have been designed to be run quickly and should take on average 5 minutes on a standard computer. The spatial and temporal resolutions of simulations varies widely. A summary of the main characteristics of the provoded samples is presented in Table 1. Each can be use as a starting template to address more complex problems.

**Table 1:** Summary of hands-on examples provided with pyBadlands package.

Exp.	dim. [km]	res.	duration	Input grids/curves & processes			
basin	30 × 30	100 m	1 Ma	rain uni.	s.l.	fluv./hillslp.	strat.
crater	2.5 × 2.5	10 m	200 ka	rain uni.		fluv./hillslp.	
delta	25 × 25	50 m	500 ka	rain uni.	s.l./tect.	fluv./hillslp.	strat.
dyntopo	300 × 200	1 km	10 Ma	rain uni.	s.l.	fluv./hillslp.	strat.
etopo	133 × 180	50 m	500 ka	rain uni.	s.l./tect.	fluv./hillslp.	
flexure	250 × 100	500 m	1 Ma	rain uni.	gflex	fluv./hillslp.	
mountain	80 × 40	400 m	10 Ma	rain oro.		fluv./hillslp.	
rift	400 × 400	2 km	250 ka	rain uni.	3D tect.	fluv./hillslp.	
strikeslip	200 × 200	1 km	100 ka	rain uni.	3D tect.	fluv./hillslp.	

abbreviations: dim.: model dimension—rain uni./oro.: uniform or orographic—res.: model resolution—fluv.: fluvial processes—hillslp.: hillslope—strat.: stratigraphic architecture—s.l.: sea-level—tect.: tectonics.

### Companion

To assist users during the pre and post-processing phases, a series of Python classes are proposed in a GitHub pyBadlands-Companion repository (https://github.com/badlands-model/pyBadlands-Companion). These classes are shipped with the Docker container mentioned in previous section. They come with IPython notebooks that have been created to illustrate how these python classes are used. We have chosen this structure to give users the transparency and opportunity to (1) clearly understand the creation and format of the input files, (2) perform quantitative analyses of pyBadlands output files, (3) easily design their own notebooks and further improve the proposed workflow.

#### Pre-processing classes

The pre-processing notebooks allows for quick creation of grids and files compatible with pyBadlands input formats. The main functionalities (and associated notebook filenames in brackets) are listed below:

- generation of topographic grids for generic model (topoCreate),
- conversion of real world topographic/bathymetric datasets into pyBadlands compatible format (etopoGen),
- building sea level fluctuations curve or using Haq’s curve [62] (seaLevel),
- generation of horizontal displacement and precipitation maps (topoTec),
- regridding of initial tectonic, rainfall and topographic input files (regridInput)

#### Post-processing classes

**Morphometric & hydrometric** The morphometrics notebook can be used to perform quantitative analyses of simulated pyBadlands landforms [3, 12]. Gradients, curvature (horizontal and vertical), aspect and discharge attributes can be extracted for the entire modeled domain or a specific area of the simulation. The hydrometric notebook allows for evaluation of time-dependent evolution of a specific catchment. It can be used to quantify the longitudinal evolution of a river profile, compute the Peclet number distribution, *χ*-maps as well as hypsometric curves.

**Fig 8:**
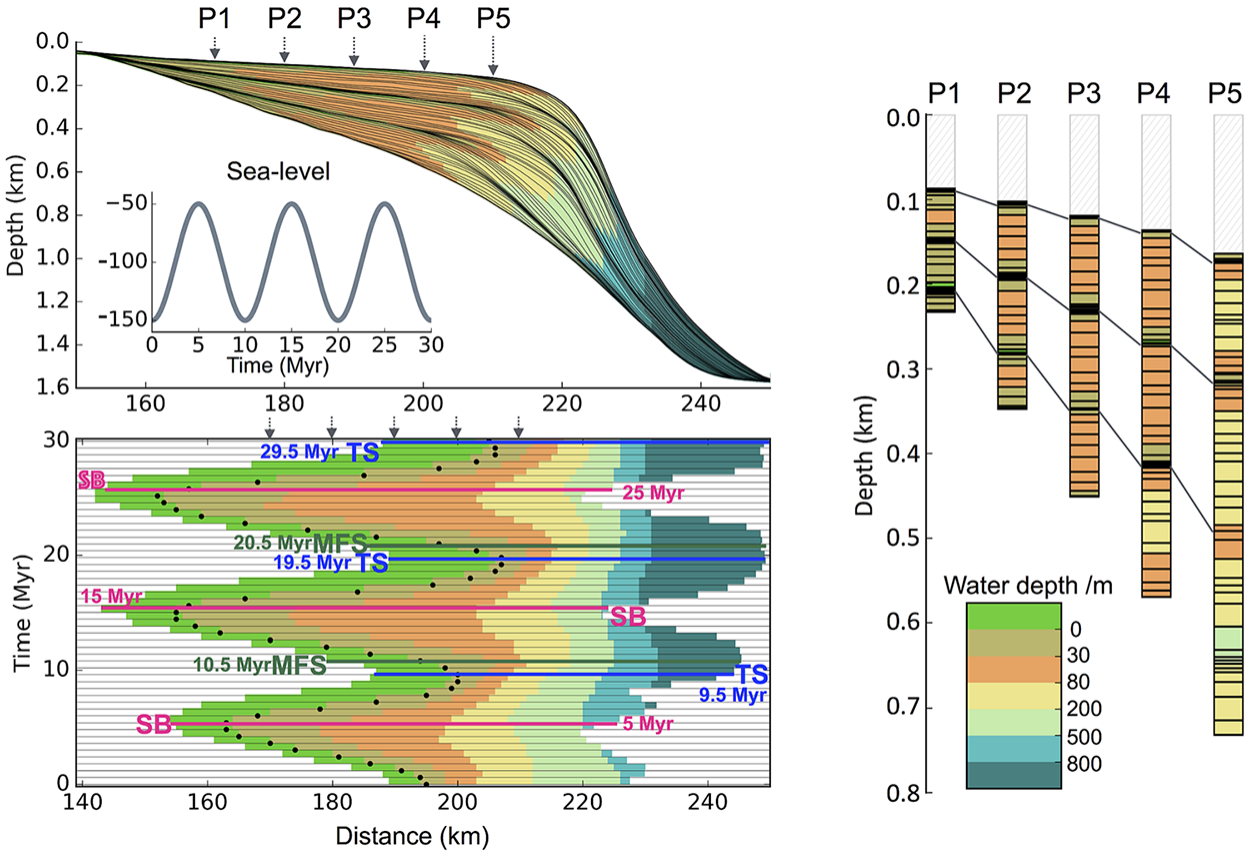
Example of stratal architecture resulting from oscillating sea level with a periodicity of 10 Ma. (a) Stratal stacking patterns on a vertical cross-section crosswise to the continental margin. Solid black lines shown on each subplot are stratigraphic layers plotted at 0.5 Myr intervals. Different colours stand for different depositional environments that are defined based on water depth (c). (b) Wheeler diagram or chronostratigraphy chart. The black dots are shoreline positions through time. The coloured lines are stratigraphic surfaces identified based on stratal terminations, stacking trends, and shoreline trajectory (SB: sequence boundaries—TS: transgressive surfaces—MFS: maximum flooding surfaces). (c) Virtual cores P1 to P5 extracted at different positions across the shelf (see location in a). Solid lines connect condensed sections and unconformities produced at low to sea-level fall.

**Stratigraphy & wheeler diagram** When the stratigraphic structure is turned on in pyBadlands, it is possible to extract cross-sections of depositional areas and plot their stratigraphic layers (Fig 8a), Wheeler diagram (Fig 8b) and virtual cores (Fig 8c). The notebook extracts simulated depositional sequences on a vertical cross-section, and calculates the relative sea level change, shoreline trajectory, accommodation and sedimentation change (Fig 8). Three methods can be applied to interpret the stratigraphic units (the conceptual framework of sequence stratigraphy) including (i) the systems tracts model based on relative sea level change; (ii) the shoreline trajectory analysis [67]; and (iii) the accommodation succession method [68, 69]. Using the stratalMesh notebook, it is also possible to export the simulated stratigraphy as a VTK structured mesh for further analysis with other software packages.

## Application to a mixed carbonate-siliciclastic system

One of the most direct applications of our model is to better constrain sediment transport processes in mixed siliciclastic-carbonate systems. To illustrate these new capabilities, we model the 14,000 years of post-glacial evolution of sediment accumulation in the central Great Barrier Reef (GBR) region and explore landscape erosion, sedimentation patterns and reef growth.

### Initial settings

#### Topography & bathymetry surfaces

**Fig 9:**
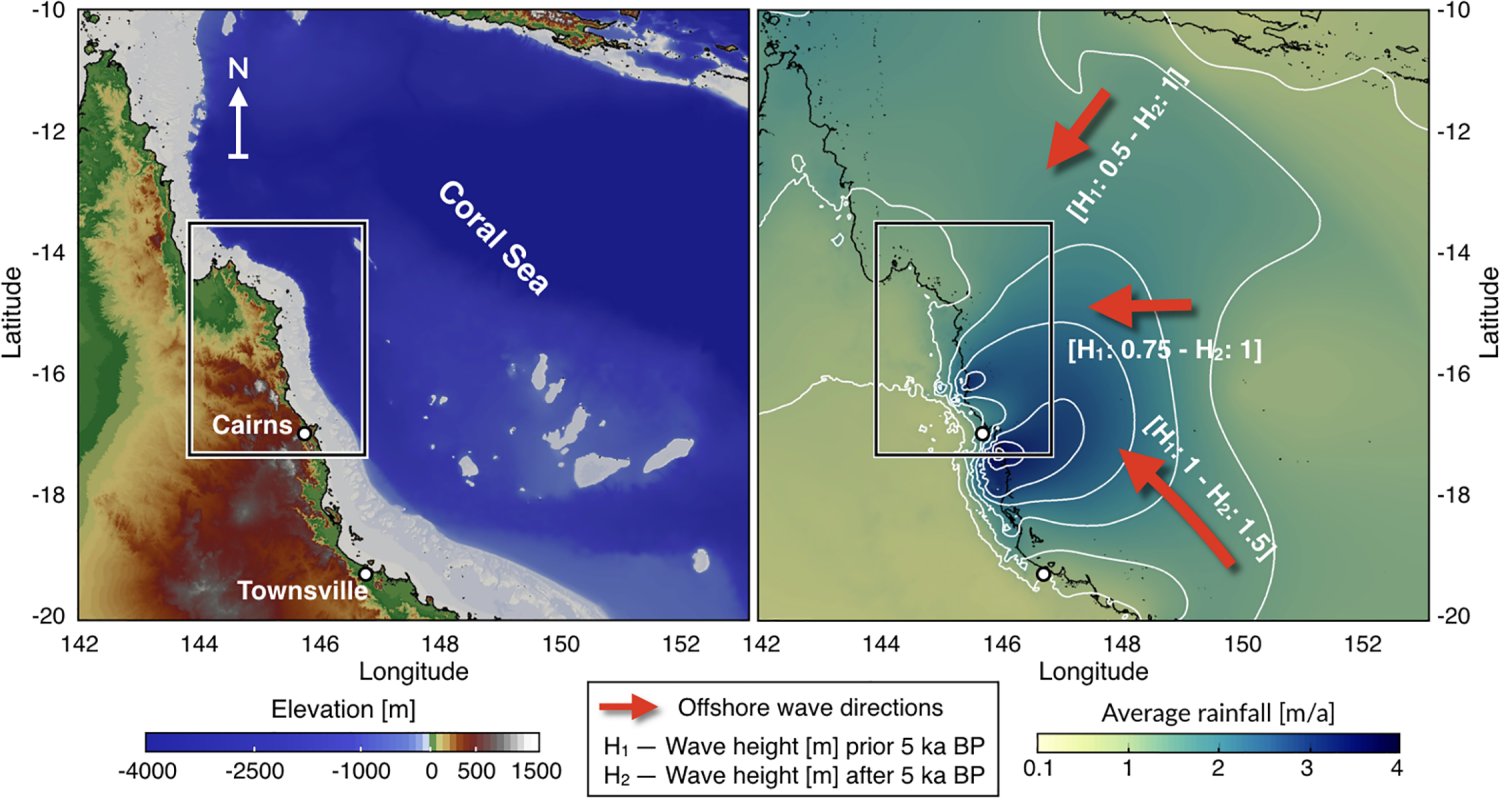
Model initial settings. Left: map shows the extend of the region of the GBR used in this example (source: Project 3DGBR—eAtlas.org.au). Right: background map shows the average rainfall annual distribution based on 30-year records (1961-1990) encompassing several ENSO events (7 El Niño—5 La Niña) (source: Bureau of Meteorology). White lines highlight precipitation 0.5 m/a contours. Red arrows define prevailing annual offshore wave directions scaled based on their annual activity. Wave heights (H) imposed for the considered 2 climatic scenarios from 14 to 5 ky and from 5 ky to present.

The initial surface used in the model is based on a 100 m resolution grid that combines high-resolution bathymetry and a land digital elevation model [70]. The dataset was resampled to a resolution of 500 m (Fig 9). To construct the paleo-surface we modified present day bathymetry to account for (1) sediment accumulation along the coast and the inner shelf and (2) coral reef development since the Holocene. Thickness of the inner shelf Holocene sediment wedge along the GBR coastline ranges from less than 5 m to 15 m [71, 72]. To construct our initial paleo-surface, we assumed an average maximum 7.5 m deposition at around the 15 m isobath, tapering to 0 m at the coast and at the 25 m isobath, and removed this estimated sediment wedge from the initial surface. The paleo-surface is then further refined by removing the average thickness of the Holocene reefs based on reef cores reaching the Pleistocene surface (reef thicknesses vary from 5 to 25 m [73, 74]).

#### Precipitation

In the region, rainfall occurs mainly during Austral summer (from November to April). Mountains along the escarpment around Cairns receive the highest rainfall, owing to orographic effects (Fig 9). Climatic reconstructions of Holocene precipitation variations generally show similar evolution attributed to the precessional control of the Walker circulation in the Pacific and the monsoon intensification [75, 76]. These reconstructions suggest three distinct periods. From the beginning of the Holocene until ∼6 ky, precipitation gradually increased. Between ∼6 to ∼4 ky, precipitation reached a maximum. Finally, from ∼4 ky to present, precipitation decreased sharply, indicative of the onset of present-day ENSO dynamics [77]. We use the 30-year average rainfall map (Fig 9) as a proxy for regional distribution of precipitation patterns. To reflect the change in deglacial and Holocene rainfall amplitudes, we scaled down today’s rainfall intensity by half at 14 ky and increased it incrementally up to 6 ky. From 6 to 4 ky, the precipitation was kept constant as 1.5 times today’s rainfall. From 4 ky to present, we imposed a linear decrease towards present day rainfall values.

#### Sea level & wave regime

The Australian region was relatively stable tectonically over the simulated period [78], therefore, sea level changes represent the main factor controlling sediment accumulation across the GBR shelf and the sea level curve from Lambeck et al. [79] is used in this simulation.

Coral Sea dominant wave direction is southeasterly and follows the trade-winds, which blow persistently in the region throughout winter [80]. In summer, a reversal of wind direction induces variable east-northeast waves and occasionally, cyclone-generated waves [72]. Holocene wave regimes are still poorly constrained for the GBR. However, observations of fossil reef cores from several locations indicate that both low and high energy corals communities have co-existed across the outer reefs since the mid-Holocene [74, 81]. Comparisons between sea level rise and reef growth also indicate that wave energies have been relatively stable over the last ∼5 ky. During early-mid Holocene time (prior to 5 ky), studies have shown that ENSO was significantly weakened and speculate that the later shift in stronger ENSO intensity was associated to an increase in wave energy. Following these observations, wave propagation in our study was forced by two climatic scenarios (Fig 9). Prior to 5 ky, offshore wave heights were distributed along the southeast [annual activity (AA): 60%, wave height (H): 1 m], east [AA: 20%, H: 0.75 m] and northeast [AA: 20%, H: 0.5 m] directions. After 5 ky, offshore wave directions followed the same pattern but wave heights increased to 1.5 m (southeast) and 1 m (east and northeast).

### Model results

**Fig 10:**
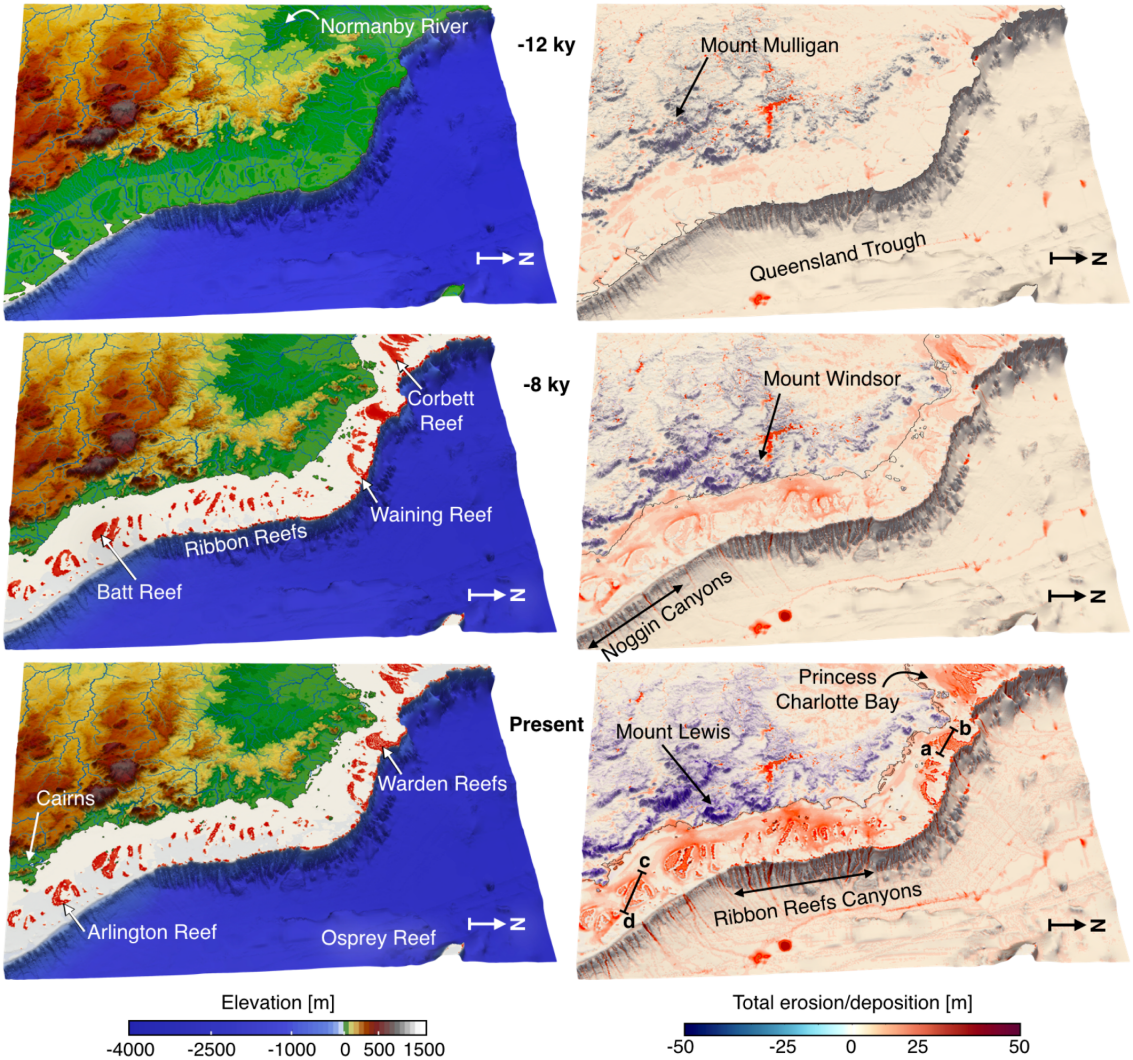
Surface evolution and simulated erosion, deposition patterns. Left: Model outputs for time steps 12 ky, 8 ky and present. Red color displays presence of coral reef at given time intervals. Right: cumulative erosion, deposition and reef evolution for the simulated 14 ky induced by the combination of fluvial and waves processes as well as reef growth.

**Fig 11:**
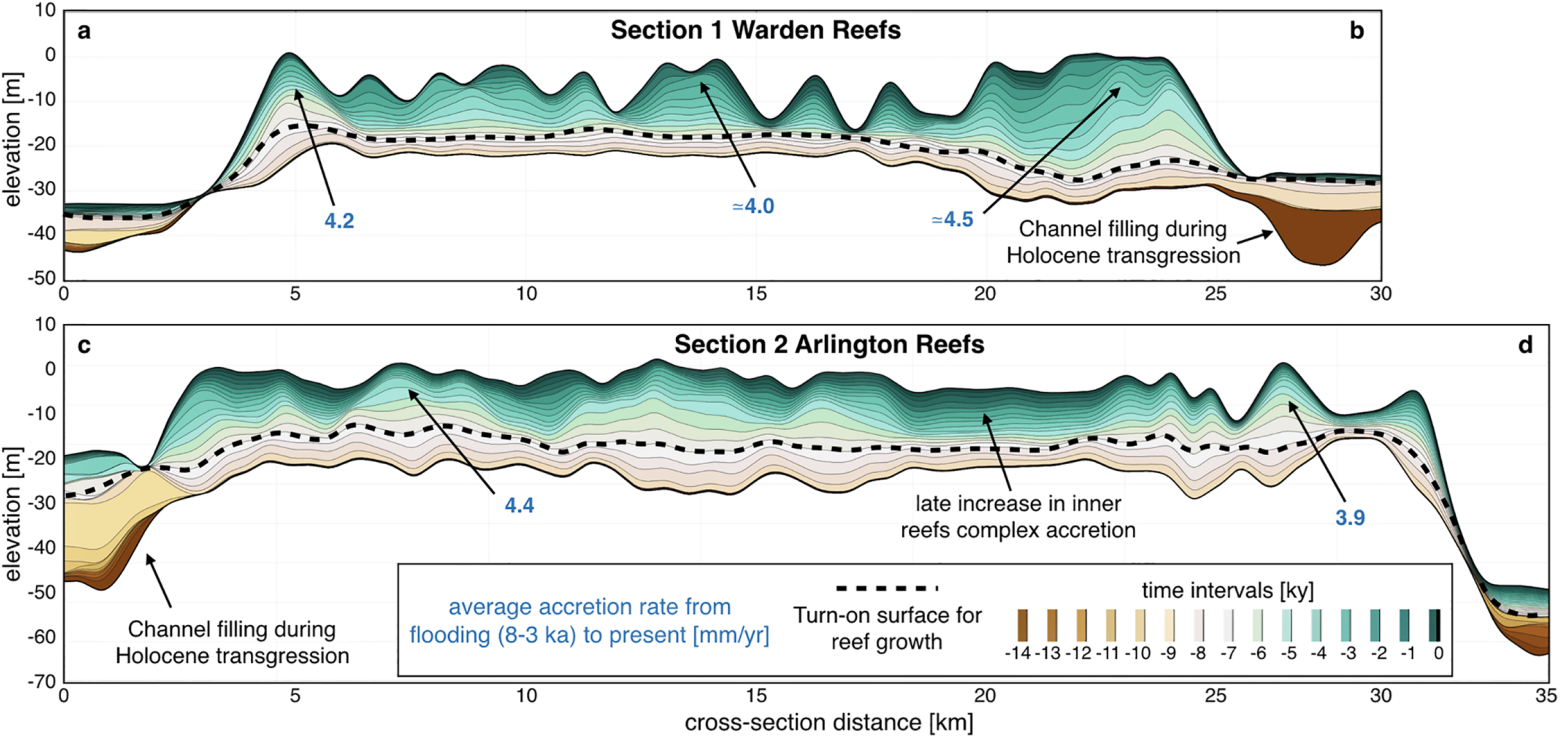
Coral reef cross-sections. Cross sections through the model predicted stratigraphy showing time layers of mixed siliciclastic-carbonate accretion across Warden Reefs and Arlington Reefs (regional locations of these sections are presented in Fig 10).

During the mid deglacial (from 14 to 10 ky), the exposed shelf acts as a bypass area where large river systems transport sediment directly to the shelf break (Fig 10). Sediment transfer on the upper slope preferentially occurs through the numerous shelf incised submarine canyons and sediments accumulate at the base of the slope and along the basin troughs. The rapid sea level transgression (prior to 8 ky) coincides with filling of previously incised channels and existing depressions (section a-b and c-d in Fig 11). Sediment transfer within the canyons persists and siliciclastic accumulation remains high in the deeper parts of the basin through the Noggin and Ribbon Reefs Canyons systems with significant accumulation along the Queensland Trough (Fig 10).

From 8 to 2.5 ky, coral reef growth across the entire shelf coinciding with decrease in rate of sea level rise (Fig 11). This period corresponds to conditions of higher rainfall and offshore wave energy; therefore, our model shows both an increase in terrigenous siliciclastic sediment delivered through riverine transport to the coast and a strong reworking of marine deposits by longshore drift on the inner shelf (bottom right panel Fig 10). A fine balance between climate, sea level and margin physiography enables coral reefs to thrive during this time interval. The rate of sea level rise is slow enough to allow, in combination with higher wave energy, active coral reef growth. As shown in Fig 11, from 8 to 3 ky, most reefs are able to catch-up with sea level rise, growing at rates faster than 4 mm/yr in some regions. The model also realistically simulates a time lag of approximately 1.8 ky between the initial flooding of the antecedent Pleistocene substrate (after 9 ky) and reef turn-on, occurring between 8 and 7 ky. We attribute this lag to the increase in sedimentation rates during the early stages of transgression. The simulated lag matches well with a documented lag of 0.7 to 2 ky after antecedent substrate flooding based on detailed radiometric dating of southern GBR drill cores [81]. Variations in reef accretion rates (Fig 11) are largely controlled by paleo-surface elevation relative to sea level position and substrate composition.

During the late Holocene, sea level stabilisation and rainfall decrease, causing reduction in fluvial erosion and marine sediment accumulation in our model. Overall reef accretion rates drop significantly over the last 3 ky as they are limited by the lack of accommodation space. Despite a general reduction in vertical accretion, we note the increase of reef accretion rates in the mid-shelf reefs complex as shown in Fig 11 (section 2). This corresponds to a phase of significant lateral reef accretion observed in a meta-analysis of all available GBR reef flat cores [82] during this time interval that also represents the later stage of reef maturity in the Hopley’s classical genetic reef model [83].

### Sediment fate from source to sink and its interactions with reefs

In our model, filling of river channels by terrigenous sediments happens quickly during the early stage of the transgression (from 14 to 11 ky as shown in Fig 11 sections). Additionally, accumulation along the Queensland Trough remains high up to the late stage of the transgression (after 5 ky, Fig 10). Current sediment supplied to the shelf is controlled by several rivers draining from mountains and tablelands adjacent to the coast (Fig 9, [84]). Our model shows that wave induced northward longshore transport redistributes: (1) terrigenous sediments in shallow water (<20 m) and (2) carbonate sediments around Holocene reefs mostly after the catch-up phase that ends around 3 ky. Sediment exchange between the shelf and the basin happens primarily through a series of shelf incised submarine canyons which morphological characteristics have influenced the Holocene sedimentation dynamics [85]. Comparisons between the Noggin (NG) and the Ribbon Reefs (RR) regions show differences in sediment gravity flows deposition with significantly higher rates in the deeper basins facing the RR region (Fig 10). This result is consistent with previous studies in the area [86], recording generally thicker and more frequent turbidites deposits in the RR canyons at this time. We found that abundant sediment gravity flows have been deposited along the Queensland Trough during the Late Holocene, consistent with available subsurface data from the ODP Leg 133 (Site 823) [87]. In our model, the depositional pattern in the trough also agrees with sonar backscatter imagery showing deep-sea sediment-laden flows following the general slope direction which gently deepens towards the north. Through the transgression, the amount of material transported to the slope continuously declines and most of the terrigenous sediments accumulate on the middle and outer shelf. After 5 ky, during the late transgression phase and the sea level highstand, neritic carbonate production (Fig 11) becomes the dominant source of sediment in the outer shelf, while terrigenous sediment is retained on the inner shelf close to the catchments outlets.

pyBadlands provides useful insights and quantitative metrics that could be used to better constrain the effects of deglacial to Holocene climatic variability on sediment dynamics in the GBR region [81, 85]. The model demonstrates that sediment accumulation is a regional geological phenomenon and has played a significant role in controlling the distribution of coral reefs during the last sea level transgression. Over thousands of years, reduction in accommodation space, due to sea level stabilisation, has generated an increase in shelf sediment accumulation. Future increase in sediment supply might result in the physical burial of inner-shelf reefs and, combined with resuspension and mobilisation of sediments by longshore drift, could also pose a significant threat to mid- and outer-shelf reefs. These predictions however will need to be balanced with projected rates of sea level rise that could increase accommodation space drastically, possibly causing: (1) restricted marine sediment accumulation to coastal domains and limited aggradation on the continental shelf; and (2) enhanced vertical reef accretion rates. Our model has the potential to quantitatively test these hypotheses in a consistent and efficient way and could be used to estimate the implications of long-term future climate predictions on the evolution of other mixed siliciclastic-carbonate systems.

## Conclusions

In this paper we present pyBadlands, an open-source python-based framework that allows for evaluation of sediment transport, landscape dynamics and sedimentary basins evolution under the influence of climate, sea waves and tectonics. It implements a wide range of physical processes which make it highly versatile and useful for applications related to source to sink problems at regional to continental scale and over geological time.

While pyBadlands has been primarily designed for research, its simplicity of use and portability should make it interesting as a teaching tool as well. Below is a summary of the main functionalities and capabilities of this framework:

- a finite volume approach from Tucker et al. [22] based on Delaunay triangulation and Voronoi diagram is used to solve the continuity equation explicitly,
- node ordering is perform efficiently based on the algorithm proposed by Braun & Willett [23],
- 3D surface deformation induced by horizontal displacements takes advantage of the node refinement technique developped by Thieulot et al. [61].
- multiple formulations of fluvial processes from detachment to transport limited cases are implemented,
- both linear and non-linear diffusion laws for hillslope processes,
- long-term evolution of longshore currents and induced sediment transport is simulated based on linear wave theory [42],
- a fuzzy logic approach is used to estimate carbonate growth based on depth, wave energy and sedimentation rate,
- orographic precipitation using Smith & Barstad [64] linear model can be used,
- varying erodibility layers could be imported to simulate their effects on landscape evolution and sediment transport.
- pre and post-processing functions allow for efficient design of new simulation, quantitative analyses of model outputs and estimation of stratigraphic evolution.

To showcase the new capabilities of pyBadlands, a simulation of the Holocene evolution of the central Great Barrier Reef is presented. It shows how our model can be used to better constrain sediment transport processes in mixed siliciclastic-carbonate systems.
